# Cancer Research

**Published:** 1904-11-05

**Authors:** 


### CANCER RESEARCH.

(*Concluded from page 91.*)

In the Bradshaw Lecture Henry Morris[3] considers the various theories of the origin of cancer. All the theories may be classed under two headings. (1) The entogenous and (2) the ectogenous. Under (1) are classed the hypotheses assigning the cause to conditions arising within the body. The principal of these are Cohnheim's "tumour germ" theory and Thiersch's "lost balance" theory. According to Cohnheim, cancer originates in misplaced embryonic cells or fœtal nests. Thiersch believes that malignant growths are started by the weakening of the connective tissues, so that the normal balance between the connective and epithelial tissues is lost, with the result that the latter grow into and destroy the former. Under (2) are placed the theories assigning the cause to conditions arising from outside the body. Such are (*a*) injury, chronic inflammation, and irritation, Virchow's theory; (*b*) Micro-organisms, either bacteria, protozoa, or fission fungi, amongst which the advocates of the parasitic theory seek the origin. None of these organisms, after years of patient study by skilled workers, have been shown to be in any sort of causal relationship to the disease. He contrasts this fact with the overwhelming evidence which Koch adduced to prove that his bacillus was the cause of tuberculosis. Some of the results of grafting and inoculation have been evoked in favour of the microbic theories, but the writer points out that the tumour products of microbic infection are the so-called granulomata, formed out of the corpuscular elements of the blood and the proliferation products of pre-existing tissue, and that all the tumours formed by the same kind of microbe are structurally the same, no matter in what part or organ they develop. But this is not the case in sarcoma or carcinoma. The secondary tumours are histologically identical with the primary, and are in no way formed out of the cells of the organ in which the metastatic growth appears. The facts prove that secondary growths are produced by the migration of cells from the primary seat. Auto-inoculation is comparable to, if not identical, with, the recognised mode of regional and metastatic infection of carcinoma, and is as unlike microbic infection as these other processes are admitted to be. He believes that Cohnheim's "tumour-germ" theory is more consistent than any other with what we know about malignant diseases, and is destined to be accepted as the true explanation of the genesis of cancer; the theory must however be extended to include groups of cells which have become isolated beyond their own natural boundaries by post-natal processes such as trauma and inflammation. That congenital matrices give rise to benign tumours, dermoid and other cysts, is generally recognised, the "tumour-germ" theory will then account for the origin of many innocent tumours, and that many innocent tumours may later become malignant is beyond doubt. Cancer very frequently arises in the situations where fœtal rests are usually found, and at the junction of different forms of epithelium, such as the lips, rectum, cervix uteri, pylorus, where zones of transitional epithelium and glands which are not functionally perfect occur. He points out that post-natal cell inclusions often occur and may be the starting points of cancer in other regions. Implantation cysts arise from the inclusion of detached epidermis in punctured wounds. Chronic inflammatory processes such as ulcers and fistulæ may often be responsible for burying detached portions of epithelial tissue. He cites 49 cases, collected from literature, of epithelioma of bone occurring at the old sites of osteomyelitis due to implantation of fragments of skin. Some of the embryonic cells during the process of repair of fractures may fail to attain the perfection of their parent cells and remain thus in a latent condition with the potentiality of becoming sarcomatous later on. That undeveloped cells may remain latent for a long period and then take on growth is shown by the cells of the organs of generation, the mammæ, the teeth, bones, etc., which for years are in a state of incomplete differentiation and then under the stimulus of puberty take on rapid proliferation. The causes which may arouse a matrix into activity are numerous; heredity, age, trauma, and chronic inflammation may all play a part. He comes to the conclusion that cancer is of local origin and is positively curable if detected and removed sufficiently early.

Plimmer[4] believes that the evidence is in favour of a parasitic origin. The alleged increase in the number of cases of late years, the grouping of cases in many instances observed in certain localities in such a way as to suggest an epidemic prevalence, the predilection for damp, low-lying, and wooded districts, and the number of records of "cancer houses" are all facts, he says, that point to a parasitic origin. The so-called cancer bodies found in the cells of malignant growths are certainly, in his opinion, parasites. The observations of those who assign different origins to them are, he considers, incorrect, and arise from the examination of fixed specimens, whereas to study these bodies it is necessary to examine fresh specimens in their own juices and upon the warm stage.

Louis Parkes[5] says that there are many *a priori* reasons for the parasitic theory. The evidence at present collected indicates very frequently an endemic prevalence. He gives an interesting account of cancer distribution in Chelsea, where a very large proportion of the total cases recorded occurred in the same streets and in groups of houses. The figures given are very striking.

A series of cases at the Middlesex Hospital have been tested with Dr. Otto Schmidt's serum.[6] A report from the investigation committee states that no trace of a specific local or general reaction to the injection was observed, and the writers feel convinced that the material is without diagnostic value. They have failed to find any improvement in the patients, or, indeed, any alteration in the course of the disease. It is claimed for the serum that :—(1) A specific reaction follows the injection, consisting of a slight elevation of temperature and general feeling of malaise; (2) a swelling in both the tumour and in any metastatic deposits or secondary glands, which then become painful, so that growths which have not been felt previously can be detected, owing to pain and swelling; (3) the reaction is of an inflammatory nature, confined to the cancerous growth, and finally arrests the growth, which becomes converted into fibrous tissue.

D'Arcy Power[7] has tested the serum on cases under his care and concludes that :—(1) The reaction was in no way specific but was such as would be obtained by the introduction of any toxin; (2) the swelling and inflammation did not occur selectively in the growth alone; it occurred in glands, which were proved afterwards to contain no growth, and in the ordinary tissues; (3) it did not appear to alter the course of the disease in any way.

Doyen[8] has prepared a toxin by means of which he claims to have obtained good results in the treatment of cancer. He uses preventive inoculations commenced soon after the removal of the disease by operation, also curative injections in cases of inoperable growths. The number of cases treated so far is 126. In 21 he reports that it is now impossible to find any trace of cancer; 18 are on the road to recovery; 29 are improved; in 58 no favourable results were noted. The nature of the serum is not divulged; he offers to send supplies to anyone desirous of trying it.

3 Brit. Med. Jour., Dec. 12, 1903. 4 Ibid. 5 Practitioner, May. 6 Lancet, March 5, 1904. 7 Brit. Med. Jour., Feb. 6, 1904. 8 Ibid., Feb. 27, 1904.

